# Sporadic and Familial Acute Myeloid Leukemia with *CEBPA* Mutations

**DOI:** 10.1007/s11899-023-00699-3

**Published:** 2023-06-01

**Authors:** Ji Yuan, Rong He, Hassan B. Alkhateeb

**Affiliations:** 1 Department of Laboratory Medicine and Pathology, Division of Hematopathology, Mayo Clinic, Rochester, MN USA; 2 Department of Hematology, Mayo Clinic, Rochester, MN USA

**Keywords:** Acute myeloid leukemia, Familial, Germline, *CEBPA*

## Abstract

**Purpose of Review:**

CCAAT enhancer binding protein A (*CEBPA*) gene mutation is one of the common genetic alterations in acute myeloid leukemia (AML), which can be associated with sporadic and familial AML.

**Recent Findings:**

Due to the recent advances in molecular testing and the prognostic role of *CEBPA* mutation in AML, the definition for AML with *CEBPA* mutation (AML-*CEBPA*) has significantly changed. This review provides the rationale for the updates on classifications, and the impacts on laboratory evaluation and clinical management for sporadic and familial AML-*CEBPA* patients. In addition, minimal residual disease assessment post therapy to stratify disease risk and stem cell transplant in selected AML-*CEBPA* patients are discussed.

**Summary:**

Taken together, the recent progresses have shifted the definition, identification, and management of patients with AML-*CEBPA*.

## Introduction

CCAAT/enhancer-binding protein alpha (*CEBPA*) is an essential transcription factor required for myeloid progenitor formation from multipotent hematopoietic stem cells [1, 2]. Mutated *CEBPA* is an important prognostic marker in acute myeloid leukemia (AML). AML with *CEBPA* mutation (AML-*CEBPA*) accounts for 5–15% of pediatric [3–7] and 7–16% of adult [8–11] AML cases, frequently in the context of a normal karyotype [12–14]. Approximately 40–50% of *CEBPA* mutations in AML are single mutation (*CEBPA*sm), and the remaining are essentially all double-mutated (*CEBPA*dm) [9, 11, 15–17]. Most AML-*CEBPA* cases are sporadic and harbor somatic *CEBPA* mutations, while approximately 10% of individuals inherit or develop de novo germline *CEBPA* mutations with predisposition to early-onset AML following acquisition of somatic *CEBPA* mutations [18].

Historically, in intermediate cytogenetic risk AML, *CEBPA*dm mutations have been associated with favorable prognosis in contrast to *CEBPA*-wild type (WT) or *CEBPA*sm, particularly those harboring the typical *CEBPA*dm pattern of concurrent N-terminal and C-terminal/basic DNA binding and leucine zipper (bZIP) domain mutations [10, 11, 15, 17]. Several recent studies have demonstrated similarly favorable outcome in bZIP-mutated cases irrespective their double or single *CEBPA*-mutated status, particularly with bZIP in-frame mutations [12–14]. These findings refine the landscape of *CEBPA* in AML risk stratification and have been adopted in the 5^th^ edition of WHO Classification (WHO-5) and International Consensus Classification (ICC)[19, 20]. In this review, we discuss the current knowledge of sporadic and familial AML-*CEPBA*, including the recent advances, and their impact on the laboratory evaluation and clinical management. We also discuss the treatment strategies for patient with *CEBPA* mutations, and the role of minimal residual disease (MRD) and stem cell transplant in this patient population.

## CEBPA Protein Structure

**Fig. 1:**
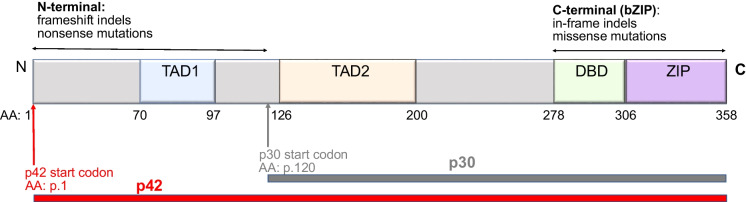
Schematic of the CEBPA protein structure and distribution of mutations. CEBPA functional domains include the N-terminal transactivation domain (TAD1), a second transactivation domain TAD2, and the C-terminal basic DNA binding (DBD) and leucine zipper domain (ZIP) (bZIP domain). The dominant p42 isoform and shorter p30 protein isoforms are translated from start codon at amino acid positions p.1 and p.120, respectively. N-terminal frameshift/nonsense mutations result in premature truncation of p42 and reinitiation of protein translation from the second start codon at p.120, leading to p30 overexpression with impaired transcriptional regulation activity. C-terminal mutations disrupt DNA binding and protein dimerization. The typical CEBPAdm mutation pattern in sporadic AML is concurrent presence of a N-terminal frameshift/nonsense mutation and a C-terminal/bZIP in-frame indel or missense mutation. In familial CEBPA-mutated AML, the dominant mutation pattern is similar to that of typical CEBPAdm, with a N-terminal germline mutation and a C-terminal somatic mutations. Indel: insertion and/or deletion

CEBPA is a member of the basic region leucine zipper family composed of six transcription factors. It is encoded by the intronless *CEBPA* gene located on chromosome 19q13.1, containing two transactivation domains (TAD1 and TAD2) in the N-terminus regulating transcription activity, and a basic DNA binding (DBD) and leucine zipper (ZIP) bZIP domain in the C-terminus responsible for DNA binding and dimerization with CEBPA protein family members (Fig. 1). By means of two alternative protein translation initiation sites in the N-terminus, *CEBPA* generates two isoforms, a full-length 42 kDa (p42) and a truncated 30 kDa (p30) (Fig. 1). The ratio of p42 to p30 appears important for myeloid differentiation and excessive p30 isoform has been shown to act as a dominant negative on the remaining p42 isoform, inhibiting the terminal differentiation of granulocytes [16, 21, 22].

## Sporadic AML with *CEBPA* Mutations

*CEBPA* mutations are observed in about 5–16% of de novo AML and can be separated into subgroups of *CEBPA*dm (50–60%) and *CEBPA*sm (40–50%). *CEBPA*dm predominantly show a N-terminal (p.1–120, transcript ID NM_004364.4) frameshift insertion/deletion (indel)-type mutation (less frequently nonsense mutation), along with a concurrent bZIP (p.278–358) in-frame indel (less frequently missense mutation). Early cloning studies have shown that *CEBPA*dm are frequently biallelic with simultaneous *in trans* occurrence of the N- and C-terminal/bZIP mutations on distinct alleles [11, 17]. A small subset of patients may harbor two *CEBPA* mutations not conforming to the typical *CEBPA*dm pattern, e.g. two N-terminal mutations, two C-terminal/bZIP mutations, one middle and one C-terminal/bZIP mutations. Rarely, homozygous single N- and C-terminal/bZIP mutations have been reported as a result of loss of heterozygosity arising from acquired uniparental disomy of chromosome19q13.1 [23, 24]. In *CEBPA*sm, mutations do not show a clear localizing pattern, and may occur in the N-terminal, C-terminal/bZIP, or middle region of the gene [10, 11, 15, 17]. *CEBPA*sm-bZIP occurs less frequently than *CEBPA*sm-nonbZIP in adults [12, 13]. Conversely, *CEBPA*sm-nonbZIP N-terminal mutations are rare in pediatric AML [3, 14]. The N-terminal frameshift/nonsense mutations enforce translation of the aberrant dominant-negative p30 isoform from the second protein translation initiation codon and thus suppress myeloid differentiation, whereas the C-terminal/bZIP in-frame indels or missense mutations disrupt protein dimerization and DNA binding. The combination of N- and C- terminal/bZIP mutations disrupt normal CEBPA function and downstream cellular processes such as cell cycle arrest and myeloid differentiation [16].

Comutation of other genes occurs more frequently in *CEBPA*sm (up to 90%) than in *CEBPA*dm, suggesting that gain of secondary mutations may be required for leukemogenesis, whereas a second *CEBPA* mutation in *CEBPA*dm may be sufficient to drive leukemogenesis without additional cooperating mutations. In a comprehensive analysis of 244 adult AML-*CEBPA*, Fasan et al. reported multiple comutations in the *CEBPA*sm cohort, including *FLT3*-ITD, *NPM1*, *ASXL1*, *IDH1/2*, and *RUNX1* with the majority (70%) of the *CEBAP*sm mutations located outside of the bZIP region [10]. Conversely, *CEBPA*dm cases were enriched with mutations of transcription factors *GATA2* and *WT1* and epigenetic modifiers *TET2* and *ASXL1*. *FLT3*-ITD and *NPM1* mutations were far less commonly seen in *CEBPA*dm [9, 11].

Clinically, *CEBPA*dm patients are associated with younger age, higher hemoglobin levels, higher WBC counts, lower platelet counts, and higher bone marrow blast percentages, compared with *CEBPA-*wild type (WT) and *CEBPA*sm cases [9, 10, 17, 25–27]. Morphologic and phenotypic findings are mostly consistent with AML with or without maturation and a subset of cases show myelomonocytic or monoblastic differentiation without significant difference seen between *CEBPA*dm and *CEBPA*sm [10, 11, 17, 28]. The blast phenotype of *CEBPA*dm is characterized by frequent expression of CD34 and some myeloid associated antigens (CD11b, CD15, CD13, CD33, CD65) with a higher frequency of CD7, CD15, and HLA-DR expression, but a lower frequency of CD56 expression than that of *CEBPA*sm [10, 29, 30]. *CEBPA*dm is strongly associated with a normal karyotype and deletion of chromosome 9 is the most frequent cytogenetic abnormality [10]. Erythroid or multilineage dysplasia is observed in a quarter of cases, though these features do not influence clinical outcomes [31].

Early studies with combined *CEBPA*sm and *CEBPA*dm cases revealed that presence of *CEBPA*m was associated with superior clinical outcomes in AML with a normal karyotype [16, 32]. Subsequent data showed that the favorable prognosis with conventional chemotherapy was restricted to *CEBPA*dm in the absence of concurrent *FLT3-*ITD in contrast to *CEBPA*sm *or CEBPA*-WT [10, 11, 15, 17, 29, 33]. Therefore, the 2016 WHO revision recognized AML with biallelic mutation of *CEBPA* as a distinct entity of AML, and AML with germline *CEBPA* mutation was placed under a separate subcategory of myeloid neoplasms with germline predisposition [34]. Both the European LeukemiaNet (ELN) and National Comprehensive Cancer Network guidelines included biallelic mutated *CEBPA* in their prognostic classifications as a favorable risk marker [35].

Interestingly and importantly, several recent studies from large-scale adult and pediatric AML cohorts demonstrated similarly favorable clinical outcome in bZIP-mutated AML irrespective their double or single *CEBPA*-mutated status (*CEBPA*-bZIP), particularly with in-frame bZIP mutations. *CEBPA*-bZIP cases also shared similar clinical and mutational characteristics with *CEBPA*dm [12–14]. Given the lower prevalence of *CEBPA*sm-bZip (1–2% of AML), previous studies may not have been adequately powered to evaluate the prognostic significance of this subset and the recent studies demonstrate the power of large-scale cohort studies in the analysis of clinical, biology, and prognostic impact of AML [12–14].

More specifically, Tarlock et al. studied the largest reported pediatric/young adult (< 30 years) AML series (*n* = 2958) and observed equally favorable clinical outcome in *CEBPA*dm and *CEBPA*sm cases harboring a bZIP mutation in comparison to *CEBPA*-WT patients, including a higher MRD-negative complete remission rate at the end of induction, identical superior 5-year event free survival (EFS) and overall survival (OS), and similarly lower relapse risk [14]. The findings were confirmed in 2 subsequent studies. Taube et al. analyzed 4078 adult AML patients and found similarly favorable OS and EFS in *CEBPA*dm and *CEBPA*sm-bZIP, but not *CEBPA*sm-TAD (with a single N-terminal mutation), in comparison to *CEBPA*-WT. When further regrouped the patients according to the presence and absence of in-frame bZIP mutations, only patients harboring in-frame bZIP mutations showed better outcome in contrast to other *CEBPA* mutations [13]. Wakita et al. reported a multicenter joint study of 1028 Japanese AML patients aged ≥ 16 years demonstrating a strong association of favorable prognosis with bZIP mutations and validated *CEBPA*dm as a cofounding factor overlapping with bZIP mutations. These recent studies also found similar clinical and biological features among *CEBPA*dm and *CEBPA*sm-bZIP cases. In the pediatric group, *CEBPA*dm and *CEBPA*sm-bZIP patients showed a comparable gene and mRNA expression profile and prevalence of cooperating mutations, including *GATA2*, *CSF3R*, *NRAS*, *FLT3*-ITD, *WT1*, and nearly absence of *NPM1* mutations. In comparison to *CEBPA*-WT, *CEBPA*dm and *CEBPA*sm-bZIP patients were older; had higher white blood cell counts; more likely to have a normal karyotype; and had a higher incidence of *GATA2* or *CSF3R* mutations, lower incidence of *NPM1* mutation, and a similar prevalence of *FLT3-*ITD and *NRAS* mutations. In adult cases, *CEBPA*dm and *CEBPA*sm-bZIP are of significantly younger age and have higher rates of CD34-positive blasts compared to *CEBPA*-WT and *CEBPAsm*-nonbZIP [12–14].

Comutations may impact on the prognosis of *CEBPA*-mutated AML. In adult *CEBPA*dm, the favorable outcome of *CEBPA*dm may be lost with a concurrent *FLT3*-ITD mutation [11, 36]. *GATA2* and *NPM1* comutations were found to be associated with superior OS, though this finding has not been consistently replicated. Similarly, possible negative impact on prognosis were found in association with *TET2*, *DNMT3A*, *WT1*, *CSF3R*, *ASXL1*, or *KIT* comutations by some but all studies [8, 10, 26, 27, 37–43]. The controversial results are likely attributable to the small sample sizes limiting the analysis power. In childhood *CEBPA*dm, *CSF3R* comutation was associated with poor relapse-free survival, but OS was not significantly different [38]. Distinct from adult AML, *FLT3*-ITD occurs with similar frequency in both *CEBPA*sm and *CEBPA*dm, and showed no effect on OS or EFS [6, 14].

In the recent large cohort studies, *GATA2* mutations were found to be enriched in both pediatric and adult patients with *CEBPA*dm and *CEBPA*sm and did not show clear prognostic significance in some studies [13, 14] while one study showed that a *GATA2*-mutated/*WT1*-wild type comutation signature carried a significantly better OS in adult patients with *CEBPA*-bZIP and an intermediate-risk karyotype [12]. *GATA2* and *CSF3R*, as well as *GATA2* and *WT1*, are mutually exclusive in *CEBPA*-mutated AML (Table 1). *WT1* mutations are frequently detected in adult *CEBPA*dm and *CEBPA*sm-bZIP, whereas *CSF3R* mutations are rare (~ 3%) in adults but are more common in children (13%) [12–14]. In pediatric study with *CEBPA*-bZIP, concomitant *CSF3R* mutations exhibited a lower EFS and a higher relapse rate with no impact on OS, confirming findings from previous studies [14, 38].

**Table 1:** Molecular genetic and pathologic features of sporadic and familial *CEBPA*-mutated AML

	Sporadic *CEBPA*-mutated AML	Familial *CEBPA*-mutated AML
*CEBPA*dm vs. *CEBPA*sm	50–60% *CEBPA*dm 40–50% CEBPAsm	Double mutations > 90%
*CEBPA* mutation loci	*CEBPA*dm: typical pattern is a N-terminal and a C-terminal/bZIP mutation; *CEBPA*sm: no localization	Most common pattern is a N-terminal germline mutation and a C-terminal/bZIP somatic mutation; C-terminal/b-ZIP or middle-region germline mutations also reported
AML subtype by morphology	FAB M1, M2, M4	FAB M1, M2, M4
Cytogenetics	Normal/intermediate-risk karyotype	Normal/intermediate-risk karyotype
Comutations	*CEBPA*dm*: GATA2*, *WT1*, *ASXL1*, *TET2*, *NRAS*; *CEBPA*sm: *FLT3*, *NPM1*, *IDH1/2*, *ASXL1*, *TET2*, *RUNX1*	*GATA2*, *WT1*, *EZH2*
Molecular profile at relapse	Relapse and evolution of the diagnostic clone	New clone distinct from the diagnostic clone

Adapted from Tawana K, Rio-Machin A, Preudhomme C, et al. Familial *CEBPA*-mutated acute myeloid leukemia. Seminars in Hematology, 54: 87–93, 2017

## Familial AML with Germline *CEBPA* Mutation

It is well recognized that myeloid neoplasms may occur in associated with inherited or de novo germline mutations. The diagnostic category of myeloid neoplasms with germline predisposition was added in the 2016 WHO classification given their increasing recognized prevalence and importance in clinical management of the patients and families [44]. Although they are rarer as compared to the sporadic myeloid neoplasms, the list likely will expand as their recognition and detection grow.

Approximately 10% of *CEBPA*-mutated AML patients harbor a germline *CEBPA* mutation [9, 18, 45, 46]. Familial AML with germline *CEBPA* mutations (FAML-*CEBPA*) generally present without preceding abnormal blood counts or myelodysplasia. The first case of FAML-*CEBPA* was described in the United Kingdom in 2004 [18, 47]. It was a family with three affected members (father, son, daughter) carrying an identical germline frameshift mutation p.P23Rfs*137 in the N-terminal of *CEBPA*. The three patients in the pedigree developed AML at the age of 10, 18, and 30 years, respectively. During the follow-up of this family, a son of the daughter harboring the same germline mutation also developed AML at an age of 2 years [18]. Akin to the sporadic *CEBPA*dm, the diagnostic AML samples from the son, daughter, and grandson all showed an additional somatic in-frame indel (duplication) mutation in the C-terminal bZIP region. Over 20 families with FAML-*CEBPA* have been reported afterwards [9, 18, 45, 46, 48–62].

In FAML-*CEBPA*, the *CEBPA* mutation pattern is mainly reminiscent of that of the sporadic *CEBPA*dm. The vast majority show a typical *CEBPA*dm pattern with a heterozygous frameshift germline mutation clustering in the N-terminus and a concurrent C-terminal/bZIP in-frame indel somatic mutation at the time of AML diagnosis. Cases with N-terminal germline mutation show near complete penetrance (90%) of AML development with typical onset at a median age of 25 years (range: 1.75–46) from a report of 10 families with 24 affected individuals [18]. Interestingly, the bZIP somatic mutations are not stable and disease relapse frequently shows a novel leukemic clone with somatic mutations distinct from the diagnostic sample, e.g., a different bZIP mutation, a second N-terminal mutation, or loss of the somatic *CEBPA* mutation [18].

Additionally, rarer FAML-*CEBPA* cases with germline *CEBPA* mutations located outside the N-terminal region have been described, involving the C-terminal/bZIP or middle region. They interestingly exhibited incomplete penetrance in contrast to the high penetrance observed in the N-terminal germline cases. In a 45-year follow-up of a large family pedigree, Pathak et al. observed a germline bZIP missense mutation p.Q311P showing an incomplete AML penetrance of 46% in 6 of 13 confirmed mutation carriers or obligate carriers. Despite the findings of attenuated DNA-binding and transactivation activities of the p.Q311P mutant in in vitro assays, seven other confirmed mutation carriers showed no evidence of hematologic malignancy with an age range of 24 to 88 years [48]. The lower penetrance of C-terminal germline mutations was also confirmed by other reports [51, 59, 63]. Additionally, three individuals were described to have germline bZIP missense mutations without a positive family history [9]. Two other groups reported a middle-region germline mutation p.E148* in 2 FAML-*CEBPA* patients with confirmed mutation carriers in the families free of hematologic malignancy at ages 37, 59, and 66. Both index patients harbored a concurrent somatic N-terminal frameshift or nonsense mutation [60, 64]. Mendoza et al. summarized the published FAML-*CEBPA* cases and compared the age of AML onset between germline mutations of different locations [64]. FAML-*CEBPA* with N-terminal germline mutations occur with the greatest frequency between ages 21 and 30 years and typically develops before age 50, whereas C-terminal germline mutations occur later with the greatest frequency between ages 51 and 60 years. The reduced penetrance and late onset of FAML-*CEBPA* patients with germline C-terminal or middle-region mutations complicate the clinical recognition of these cases and likely contributed to its apparent rarity and probable underestimation. Therefore, the persistence of a *CEBPA* mutation at the time of complete remission warrants further germline testing, irrespective of family history.

Based upon the cohort with N-terminal germline mutations, the clinicopathologic and molecular characteristics of FAML-*CEBPA* were found to resemble those of its sporadic counterpart [18]. They are commonly associated with FAB subtypes M1 and M2, with a normal karyotype, and similar coexisting mutation profile including *GATA2*, *WT1*, *EZH2*, *TET2*, and *NRAS* mutations. Affected family members were observed to share almost identical comutations, indicating that germline *CEBPA* mutations may influence the acquisition and selection of specific cooperating mutations, which may be governed by the inherited genetic factors shared within families. FAML-*CEBPA* is associated with favorable long-term outcomes with a reported 10-year OS of 67%, but a higher relapse incidence (50–90% in different series). Multiple relapses may occur and can span decades. The first relapse usually presents later than sporadic cases. Median time to first relapse in FAML-*CEBPA* and sporadic ones were reported to be 27 months, and 11 months, respectively. Superior response to secondary therapies has been observed with a median survival of 8 years in contrast to 16 months in sporadic *CEBPA*dm following recurrence [18, 45, 65]. The genetic landscape and clinical heterogeneity of FAML-*CEBPA* are evolving with the more recent findings of cases with non-N-terminal germline mutations showing lower penetrance and longer latency. Long-term follow-up of these patients and symptomatic carriers would allow better characterization of the true prevalence and clinical and genetic heterogeneity of FAML-*CEBPA* and ultimately enhance patient care.

## Laboratory Evaluation of *CEBPA* Mutations

With the wide breath of *CEBPA* mutations spanning from N-terminus to C-terminus and multiple mutation types (point mutations and indels), the most comprehensive method for *CEBPA* mutation detection is full length sequencing of this single exon gene. Before the wide clinical use of next-generation sequencing (NGS), it is commonly done by Sanger sequencing. As NGS has become routine in AML diagnostic work-up, *CEBPA* is frequently included in NGS panels which offer the convenience of simultaneous interrogation of multiple clinically informative genes. Sanger sequencing has a well-established analytical sensitivity of 15–20% whereas the sensitivity of many NGS panels is approximately 5%. Both platforms performing at the expected sensitivity are adequate to detect a germline mutation in the absence of events that may alter the variant allele fraction (VAF) such as concurrent copy number variation of the genetic region or allogenic stem cell transplantation. NGS can additionally detect somatic *CEBAP* mutations with lower VAF falling below the analytical sensitivity of Sanger sequencing. One technical caveat is that the *CEBPA* gene is GC-rich, necessitates vigorous validation and optimization during the test development phase to ensure reliable clinical performance. Capture-based NGS has been shown to perform better than amplicon-based NGS in *CEBPA* mutation detection [66]. As indel-type mutations are frequent in *CEBPA*, in medical facilities with limited resources for complex molecular testing, fragment analysis may be used as a screening tool to identify indel type mutations [3, 67]. This method usually offers an analytic sensitivity around 5%; however, it will not detect point mutations or offer precise sequence information of the indel events. Furthermore, with the precision limitation of current capillary electrophoresis platforms, fragment analysis does not offer the capability of precisely determine the indel size (and therefore lack the ability to differentiate between in-frame and out-of-frame indels) as the indel sizes increase. Historically, some labs also used denaturing high-performance liquid chromatography to screen for point mutations and small indels in *CEBPA* followed by sequencing in cases with abnormal chromatograms [11].

One important aspect of *CEBPA* testing is follow-up germline mutation confirmation in AML-*CEBPA* patients. This information is important for patient management including allogeneic stem cell transplant donor selection as well as patient family consultation and surveillance. In a diagnostic peripheral blood (PB) or bone marrow (BM) sample, typically a heterozygous germline variant in a treatment-naïve patient would show a VAF around 50%. However, VAF cannot be reliably used as a marker to differentiate between germline versus somatic events in PB or BM as a VAF around 50% can also be seen in somatic settings and the VAF may be altered by other concurrent events such as copy number variation, loss of heterozygosity, and allogenic stem cell transplant. In AML patients undergo intensive chemotherapy, a strong clue for the presence of a germline *CEBPA* mutation is that at the time of complete remission, germline mutation persists (with a VAF around 50% similar to the diagnostic sample) while the other somatic mutation disappears [10, 11, 60]. For hematologic malignancies, PB or BM samples are not the appropriate constitutional sample type for germline testing in hematologic malignancies. The gold standard constitutional sample type for germline confirmation is cultured skin fibroblasts, with the caveat that the procedure is invasive and labor intensive. Other germline sample types include hair follicles, purified T-lymphocytes, saliva DNA, and buccal swab [9, 10, 18, 68]. The rare occurrence of revertant somatic mosaicism seen in Fanconi anemia and dyskeratosis congenita has not be reported in FAML-*CEBPA*[69, 70]. Family member testing with normal blood counts maybe be performed in PB DNA. However, these results should be verified with constitutional DNA, particularly in allogeneic stem cell transplant donor candidates. Germline genetic testing has profound psychosocial impacts, and the importance of multidisciplinary support and genetic counseling cannot be overstated.

Although biallelic mutation is required for AML with biallelic mutation of *CEBPA*, it is technically impossible for either Sanger sequencing or routine (non-long-range) NGS to definitively delineate whether the concurrent N- and C-terminal mutations are biallelic, as they are far apart on different amplicons (Sanger sequencing) or sequencing reads (by NGS), and may reflect true biallelic events occurring on separate alleles (in trans) in the same cell, or two mutations involving the same allele (in cis), or distinct occurrences in separate subclones. However, earlier cloning analysis has shown that the typical *CEBPA*dm pattern of concurrent N- and C-terminal/bZIP mutations is highly likely to represent a true biallelic mutation event [11, 17]. In clinical practice, there are rarer occurrences of *CEBPA*dm not conforming to the typical *CEBPA*dm pattern with various combination of mutations occurring in N-, C- or middle regions of the gene. The clinical outcome of these cases has not been thoroughly studied in large cohorts, although few groups observed distinct methylation and gene expression patterns from the typical *CEBPA*dm and a trend toward worse OS particularly after relapse [40, 71]. These findings add complexity and confusion to the precise assignment of “AML with biallelic mutation of *CEBPA*” in daily clinical practice.

Rather than the previous requirement for biallelic *CEBPA* abnormalities, the recent finding of favorable outcome of in-frame bZIP-mutation irrespective of double or single *CEBPA*-mutated status [12–14] has been adapted in both ICC and the 2022 ELN recommendation, while WHO-5 continues to include both *CEBPA*dm or bZIP *CEBPA* mutation[19, 20, 72]. In addition, ICC and ELN require a minimum of 10% blast for AML-*CEBPA*, whereas WHO-5 mandates 20% blast threshold. Hopefully, a consolidated definition of AML-*CEBPA* would be agreed upon among the WHO, ICC, and ELN in the near future to clarify the diagnosis and prognosis of this entity.

## Treatment

In general, AML-*CEBPA*dm is a chemosensitive disease with a high response rate. The anthracycline and cytarabine based induction chemotherapy followed by consolidation remain the main therapy for this favorable risk AML in medically fit patients. Although the gemtuzumab ozogamicin has improved outcomes of cytogenetic favorable risk AML, its role in molecularly favorable AML like *CEBPA*dm remains unclear [73]. Recently, hypomethylating agents (HMA) coupled with venetoclax have been adapted as frontline therapy for patients who are not eligible for high intensity chemotherapy; however, favorable risk AML was excluded from the VIALE-A phase III randomized trial [74]. Arslan et al. studied the use of HMA + Venetoclax in favorable risk AML [75]. Thirteen (30%) out of 43 patients had AML-*CEBPA*dm. The complete remission (CR) and complete remission with incomplete count recovery (CRi) rate in this patient population were 75%.

MRD assessment is currently available for clinical practice. Deng et al. evaluated the role of multiparametric flow cytometry (MFC) MRD in *CEBPA*dm AML-*CEBPA*dm [76]. MRD-positive status during consolidation but not after induction was associated with an increased risk of relapse and decreased relapse free survival. However only elevated WBC count at time of diagnosis was prognostics of these outcomes in multivariate analysis. Wang et al. further stratified MRD as low risk and high risk MRD [77]. The low risk MRD was defined as negative MRD after at least two consolidation cycles of chemotherapy. Low risk MRD was not only associated with a lower risk of relapse, and improved relapse free survival (RFS) but was also associated with improved OS.

On the other hand, several early studies have identified the limited role of stem cell transplant in CR1 as it was not associated with improved OS [78]. Schlenk et al. studied the role of stem cell transplant in CR1 in the absence of MRD data. While both autologous and allogeneic stem cell transplant provide improved RFS in CR1, the OS was not different compared to patient who received chemotherapy only. This study provided evidence to reserve hematopoietic stem cell transplant to CR2 or refractory disease.

Relapsed AML-*CEBPA*dm continues to retain chemotherapy sensitivity. The second CR rate was reported to be 83–85% [78, 79]. Wang et al. reported that patients who are in second CR or have refractory disease, benefited of allogeneic stem cell transplant [77]. Yet allogeneic stem cell transplant remains an important consolidative therapy in the prevention of future relapses in patient FAML-*CEBPA*, as it can treat the AML in addition to replace leukemia prone stem cells [53]. This highlights the importance of careful germline screening for patient with suspected FAML-*CEBPA*, as it would have implications on matched sibling donor selection since donor derived AML-*CEBPA* has been reported in the literature [80].

## Conclusion

Here we reviewed the current knowledge of *CEBPA* mutation, co-mutation, and the recent advances and recognition of the favorable outcome of bZIP-mutation and its incorporation to the WHO-5, ICC, and ELN classifications. We also provided a detailed laboratory evaluation for *CEBPA* germline mutation for patient with suspected FAML-*CEBPA*. We elaborated on the current available therapies, the implications of MRD in stratifying the disease risk post therapy, and the role of stem cell transplant.

While AML-*CEBPA* generally harbors a favorable prognosis, further studies are required to address best strategies for the treatment of MRD-positive disease, and how to improve the outcomes of co-mutated *GATA2*, *WT1*, and *CSF3R* AML.
